# Takotsubo syndrome: between evidence, myths, and misunderstandings

**DOI:** 10.1007/s00059-020-04906-2

**Published:** 2020-03-23

**Authors:** L. Christian Napp, Johann Bauersachs

**Affiliations:** Department of Cardiology and Angiology, Hannover Medical School, Carl-Neuberg-Str. 1, 30625 Hannover, Germany

**Keywords:** Stress cardiomyopathy, Acute heart failure, Cardiogenic shock, Mechanical circulatory support, Pulmonary artery catheterization, Stresskardiomyopathie, Akute Herzinsuffizienz, Kardiogener Schock, Mechanische Kreislaufunterstützung, Pulmonalarterienkatheter

## Abstract

Takotsubo syndrome (TS) is an acute cardiac condition characterized by transient wall motion abnormalities mostly of the left ventricle. First described in 1990, TS has gained substantial attention during the past 15 years. However, the disease is still underdiagnosed. Prospective studies on TS are largely lacking, and the condition remains incompletely understood. In addition, significant misconceptions and misunderstandings are evident, contributing to potentially severe underestimation. Here, we review important aspects of TS with a focus on pitfalls, misinterpretations, and knowledge gaps considered important during diagnosis and management of the disease.

**Electronic supplementary material:**

The online version of this article (10.1007/s00059-020-04906-2) contains the references to the tables. The article and additional material are available in the electronic full text archive at http://www.springermedizin.de/herz. You will find the additional material at the end of the article under “Supplementary Material”.

## Abstract (de)

Das Takotsubo-Syndrom ist eine akute Erkrankung, die durch transiente Wandbewegungsstörungen überwiegend der linken Herzkammer gekennzeichnet ist. Nachdem sie 1990 erstmals beschrieben wurde, hat sie insbesondere in den letzten 15 Jahren enorme Aufmerksamkeit erfahren. Dennoch ist die Erkrankung unterdiagnostiziert. Es gibt kaum prospektive Studien, und das Takotsubo-Syndrom ist in vielen Aspekten noch nicht ausreichend verstanden. Darüber hinaus existieren zahlreiche Missverständnisse, die potenziell dazu beitragen, die Erkrankung zu unterschätzen. Hier geben die Autoren einen Überblick über wichtige Aspekte des Takotsubo-Syndroms unter besonderer Berücksichtigung möglicher Fehlinterpretationen und Wissenslücken, die für Diagnose und Behandlung von Bedeutung sind.

Takotsubo syndrome (TS) is an acute cardiac condition originally described in 1990 in a book chapter [1] and in 1991 in a Japanese journal article [2]. Of note, several publications before 1990 reported patients who very likely had TS [3], however, without using the name Takotsubo. Most TS patients suffer from rather acute chest pain and dyspnea, and about two thirds of patients have experienced a preceding trigger, which may be either an emotional event such as anger or grief or a physical incident such as trauma, surgery, or infection, or both [4]. Since 50% of patients have ST-segment elevation on the ECG and cardiac biomarkers are usually elevated to a relevant extent, many TS cases are diagnosed by cardiac catheterization originally performed for suspected myocardial infarction [5]. TS is characterized by transient wall motion abnormalities with hypo- or more often akinesia of midventricular and apical segments of the left ventricle (LV) as well as hypercontractile basal segments. However, atypical forms exist involving only midventricular, basal, or focal parts of the LV, constituting about 25% of cases. Left ventricular ejection fraction (LVEF) is often severely reduced, and LV end-diastolic pressure (LVEDP) markedly elevated, both of which reflect acute impairment of systolic and diastolic LV function [4, 6]. A hallmark of TS is an often rapid recovery of wall motion abnormalities within days to weeks, which needs to be demonstrated by imaging in order to finally diagnose the disease, unless the patient dies beforehand [7]. Taken together, TS represents a prototypical acute heart failure syndrome, although the initial clinical presentation mimics that of an acute coronary syndrome.

While TS remained largely unnoticed for approximately 10 years after the initial description, the condition has gained enormous attention over the past years (Fig. 1). TS is still considered to be underdiagnosed, with an underestimated risk and incompletely understood pathogenesis [8]. Importantly, numerous misunderstandings have emerged in the context of TS. This is in part due to a lack of knowledge resulting in unproven assumptions, and in part due to some erroneous messages from early reports. However, there is also a phenomenon of “ghost messages,” which are repeatedly re-featured in reviews, letters, and sometimes also original studies, despite already convincing evidence from the existing literature. In addition, owing to the relatively low incidence of the condition, a large number of single case reports was published massively outnumbering original studies (Fig. 1). This was very likely associated with a reporting bias, as predominantly “clear” cases were published that were in line with early reports. Overall, a misconception of the disease has evolved: TS is still widely considered a benign, transient, “self-healing” disease with an emotional trigger and “clean” coronary arteries, but without relevant complications. In clinical routine one can even hear opinions such as, “I suspected my patient was suffering from acute myocardial infarction, but after all it was only Takotsubo,” reflecting a significant underestimation. Already the title of the first official description (“Takotsubo-type cardiomyopathy due to multivessel spasm”) [1] contained the term “cardiomyopathy,” suggesting a rather chronic condition, for which no robust evidence exists. In contrast, TS is not a benign disease [9], is not uniformly preceded by an emotional trigger [4], and does not require “clean” coronary arteries (see below). Based on the available evidence on TS, the present review focuses on pitfalls, misinterpretations, and knowledge gaps considered important during diagnosis and management of the disease.

**Fig. 1:**
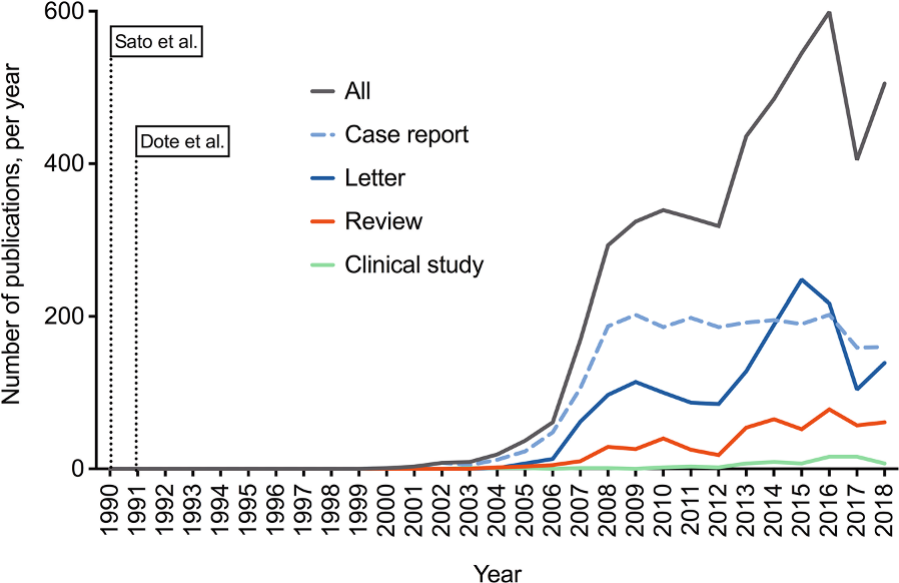
Medline-listed publications on Takotsubo syndrome over time. PubMed entries per year, for the search term “Takotsubo.” *Case report*, *letter*, *review*, and *clinical study* refer to publication types as predefined by PubMed. Search was performed using an online search tool [135]

## Nomenclature

Most newly described entities or therapies undergo a change in their name or abbreviation. Indeed, TS has been ascribed numerous different names, especially in early years. While the initial description used the term “Takotsubo-type cardiomyopathy” [1], many of the following publications used “Takotsubo cardiomyopathy,” “left ventricular apical ballooning syndrome,” “broken heart syndrome,” or “stress cardiomyopathy” instead, among others (Table 1). However, these names either suggest that only the left ventricle is affected, or that the heart is always “broken” (emotional trigger), or that TS is a cardiomyopathy, all of which are not generally true. In 2011, an analysis of published reports uncovered that already at that time, 75 individual names had been used for the same condition [10]. These names were accompanied by an equally confusing number of abbreviations. Accordingly, a substantial debate on the nomenclature emerged [10–16], which was also related to different diagnostic criteria (see below). In recent times, most scientists and TS experts agree to use the term “Takotsubo syndrome,” abbreviated either as “TS” or “TTS.” The word “Takotsubo” is a metaphor for the apical type of TS, where the shape of the LV during systole resembles a pot (Jap. tsubo) used to trap octopods (Jap. tako) in Japan. Even though it is not intuitive to use it for atypical TS types, the metaphor takotsubo prevents confusion and appreciates the pioneering work of Japanese researchers [10]. While some reports used a hyphenated version (“Tako-tsubo”), the nonhyphenated version is much more frequently used: Until 2 January 2020, Medline contained 616 publications with “Tako-tsubo” as a title word and 3062 publications with “Takotsubo” in the title.

**Table 1:** Takotsubo synonyms

Synonym (abbreviation)
Ampulla cardiomyopathy
Apical ballooning syndrome (ABS)
Left ventricular apical ballooning syndrome (LVABS)
Transient apical ballooning syndrome
Transient cardiac ballooning
Transient left ventricular apical ballooning (TLVAB)
Takotsubo cardiomyopathy (TC, TTC, TCM)
Takotsubo-like cardiomyopathy
Takotsubo cardiomyopathy syndrome
Takotsubo stress cardiomyopathy
Takotsubo syndrome (TS, TTS)
Stress cardiomyopathy (SCM)
Stress-induced cardiomyopathy
Broken heart syndrome

An overview of many more and also rarely used names for TS has been published elsewhere^S1^

References to the tables are provided as electronic supplementary material

Documentation of recovery of TS-related wall motion abnormalities, i.e., normalization of systolic LV function in most patients, is required to diagnose TS across all types of diagnostic criteria (see below). Indeed, the often fast recovery of systolic LV function is a hallmark of TS and frequently astonishes treating physicians who had just encountered severe systolic dysfunction in their patients. This led to the assumption that TS is a “transient” disease and contributed to the misconception that the associated risk would be very low. Although there is new evidence that a TS episode results in subtle myocardial damage (see below), the term “cardiomyopathy” seems inappropriate [12] since to date there is no proof that *relevant* myocardial damage occurs. In contrast, the term “syndrome” better describes a condition that is incompletely understood, is probably not only a cardiac disease, and occurs in different settings. To date, it still remains unclear whether TS is a cardiac or extracardiac disease. The substantial incidence of TS in patients with extracardiac conditions such as pheochromocytoma, acute cerebral pathologies, and after administration of sympathomimetic drugs suggests that TS could be an end-organ epiphenomenon of an extracardiac disorder. Thus, overall it seems most meaningful to generally use the term “Takotsubo syndrome” for all typical and atypical TS forms.

## Spasm, catecholamines, gender, and the brain

Initially, TS had been associated with macrovascular coronary spasm, as already reported in the first description of the disease [1, 2]. Several authors described either spontaneous or provoked spasm in TS patients [17]. However, wall motion abnormalities in TS are usually not congruent with the perfusion territory of an epicardial coronary artery, and therefore it appears rather unlikely that spasm in larger arteries is the cause of TS. This, however, does not exclude that spasm in smaller vessels, i.e., in arterioles, would be a key step during the development of TS.

In 2005, a pioneering study found higher levels of circulating catecholamines in TS patients as compared with age- and gender-matched patients with ST-segment elevation myocardial infarction (STEMI; [18]), suggesting that TS might be induced by a catecholaminergic surge. Although the sample size in that study was rather small, it gave the initial spark for research and recognition of the disease, which lasts until today (Fig. 1). However, subsequent studies had conflicting results [19–21], leaving the question of whether or not TS is essentially associated with increased levels of circulating catecholamines largely unanswered. Thus, the debate on circulating catecholamine levels is ongoing [22]. Still, local myocardial catecholamine effects may be essential for stunning irrespective of circulating catecholamine levels. Many case reports have been published describing TS onset early or immediately after catecholamine administration [23]. However, compared with the high number of patients receiving catecholamines worldwide, the incidence of TS is still inadequately low, rendering catecholamine administration a trigger rather than a causative step. It is tempting to assume that coronary arteriolar spasm mediated by catecholamines induces TS. Importantly, spasm may also be a symptom demonstrating the catecholamine surge, i.e., rather be an epiphenomenon. The Mayo Clinic Diagnostic Criteria excluded pheochromocytoma as a cause of TS [24, 25], but there is no characteristic phenotypic difference between apical ballooning in pheochromocytoma and “classic” apical TS. The TS-like acute cardiac dysfunction in patients with pheochromocytoma is therefore no longer considered a different condition [7]. It should instead be considered a secondary form of TS, with pheochromocytoma as the trigger [16]. In turn, coexisting pheochromocytoma should be remembered and ruled out in TS patients with hypertensive emergency or shock.

Generally, some kind of sympathetic surrounding, either of endogenous or exogenous origin, is considered essential during TS development. However, this does not necessarily require an identifiable “stressor.” If an emotional or physical trigger is not present despite extensive history taking, there may still be a hidden source of stress or an unnoticed sympathetic surge. Notably, it is unknown how the characteristic wall motion abnormalities in TS develop. An animal study with rats has proposed a model in which differential beta-receptor signaling in basal and apical cardiomyocytes is responsible for the apical TS phenotype [26]. The concept behind this study appears very attractive for explaining the coexistence of basal hypercontraction and midventricular and apical hypo- and akinesia in the same heart. However, atypical forms of TS cannot be explained with this model, and there are many published reports of patients who had two, three, or even more episodes with different TS forms over time (see below), practically excluding that the proposed model is the primary explanation for the cardiac TS phenotype. Overall, albeit there is a “typical” association, from a mechanistic point of view catecholamines and sympathomimetic substances can neither be considered essential nor sufficient for TS development, and the debate is ongoing.

TS has also been proposed to be a special form of acute coronary syndrome (ACS). Troponin levels are elevated in virtually all TS patients, although levels are usually inappropriately low compared with the often severe systolic dysfunction of the LV. TS is characterized by the absence of substantial late gadolinium enhancement on cardiac magnetic resonance imaging (MRI, see below), which is an essential difference to ACS [27]. Therefore, ischemic myocardial necrosis does not explain wall motion abnormalities in TS. Of note, myocardial dysfunction may also be caused by ischemia without occurrence of necrosis, as ischemia is generally able to result in severe stunning of the heart [28]. Nuclear tests support this hypothesis, as perfusion is moderately reduced but metabolism strongly impaired in stunned myocardial segments in TS [29].

Occasionally, TS has been reported to occur in close relatives or siblings [30, 31]. Although TS is supposed to be nonhereditary, it is very likely that a genetic basis for susceptibility to triggers and sympathetic surroundings exists [32]. This preexisting vulnerability would further explain, at least in part, the recurrence of TS. The anecdotally described concurrent onset of TS in close relatives illustrates that there must be some biological background that in conjunction with an external trigger finally results in development of TS. Genetic studies have already identified promising loci, copy number variations, and polymorphisms in TS patients [33–39]; however, these results require further confirmation and exploration before allowing for mechanistic conclusions.

Another key toward understanding the pathogenesis of TS might be hidden in the striking gender preponderance: 90% of patients are women, and of those 80% are postmenopausal. This led to the hypothesis that estrogen is a relative protective hormone, and that its decline may predispose individuals to TS development. In rats, estrogen supplementation attenuates the cardiac phenotype of immobilization stress [40, 41], and estrogen levels in patients with subarachnoid hemorrhage and LV dysfunction are lower than in those with normal systolic LV function [42]. Estradiol protects cardiomyocytes from isoproterenol-induced ROS-production and action potential duration prolongation [43]. In a recent study, women with TS and women matched for age and gender with STEMI had comparable levels of several sex hormones [44]. However, this study lacked a healthy control group. Furthermore, it is questionable whether measurement of circulating sex hormones at a single timepoint during the acute phase of the disease sufficiently reflects the complex sex hormone network. An earlier study found lower estradiol levels in TS patients than in STEMI patients or healthy controls [45]—acutely as well as at 6 years’ follow-up. Interestingly, glucose metabolism in the adult heart has strong sex preferences [46], which points toward cellular metabolism as an important sex-dependent factor during TS pathogenesis. Overall there remains much room for further research in order to understand the gender differences in TS.

TS is not rare in patients with acute neurological conditions, and in turn the prevalence of neurological disease in TS is higher than expected by random chance. In total, 50% of patients with TS suffer from an acute or chronic neurological or psychiatric disease [4], which suggests that the brain may be a critical component during pathogenesis. A recent study showed characteristic activity of brain regions in functional MRI [47], which may be the basis for future research to identify specific changes of regional brain function potentially inducing myocardial stunning. As outlined above, a “humoral” hypothesis postulates that circulating catecholamines, sex hormones, and others finally induce or trigger TS. Interestingly, reports of TS in patients after heart transplantation are extremely rare [48–50], supporting the notion that an anatomical brain–heart axis may usually be required for development of TS. Overall, there is much room for innovative studies on the interaction of brain and heart in TS.

## Age and trigger

Initially TS was mainly reported in postmenopausal women. Indeed, most patients with TS are female. In large registries the mean age is approximately 68 years, with 90% being women. However, over time many cases of TS were reported in men, and also in very young [51] and very old patients (Table 2). The youngest reported patient is a 9-day-old preterm infant, and the oldest one a 101-year-old woman. This demonstrates that TS should be considered a potential diagnosis across all genders and ages.

**Table 2:** The age spectrum of takotsubo syndrome

Study	Year	Sex	Age	Reported trigger	TS type
Rozema et al.^S2^	2016	Female	9 days (born premature at 27.6 weeks’ gestation)	Unknown	Apical
Greco et al.^S3^	2011	Male	2 days	Fetal distress, hypoxia	Apical^a^
Hernandez et al.^S4^	2010	Male	16 months	Cyclic vomiting	Apical
Schoof et al.^S5^	2010	Female	2 years	Surgery	Apical
Maruyama et al.^S6^	2006	Female	32 months	Buprenorphine and midazolam withdrawal	Apical
Fabi et al.^S7^	2013	Female	4 years	Abdominal pain, fever, diarrhea	Apical
Otillio et al.^S8^	2014	Female	6 years	Hemophagocytic lymphohistiocytosis	Apical
Berton et al.^S9^	2012	Female	12 years	Spinal surgery, resuscitation	Apical
Srivastava et al.^S10^	2016	Male	14 years	Prolonged seizure, after 6 months TS recurrence, again after seizure	Apical
Zalewska-Adamiec et al.^S11^	2018	Female	15 years	Emotional distress	Apical
Ohwada et al.^S12^	2005	Female	17 years	Hypoglycemia, anorexia	Apical
Okwechime et al.^S13^	2018	Female	26 years	K2 synthetic marijuana abuse	Apical
An estimated 70% of patients are postmenopausal women with a mean age of 65–70 years					
Singh et al.^S14^	2012	Female	89 years	Grief	Apical
Xu et al.^S15^	2012	Male	90 years	Pneumonia	Apical
Budnik et al.^S16^	2015	Female	98 years	Chronic emotional stress	Apical
Zalewska-Adamiec et al.^S17^	2016	Not reported	100 years	Not reported	Not reported
Bonfanti et al.^S18^	2018	Female	101 years	Emotional distress	Apical

References to the tables are provided as electronic supplementary material

*TS* takotsubo syndrome

^a^Likely apical TS, but TS type cannot be finally determined from the reported information in this case

From the beginning, TS was reported to be preceded by emotional triggers such as anxiety, fear, depression, or grief. Later on, awareness of physical triggers such as sepsis, trauma, surgery, acute intracerebral conditions, respiratory distress, bleeding, or diarrhea emerged. Importantly, emotional triggers may also be positive ones such as immense joy or achievements, also termed “happy heart syndrome” [52]. Of note, in some patients a physical trigger also serves as an emotional one, e.g., when physical trauma is associated with pain and fear, and in these patients it is virtually impossible to distinguish the finally leading trigger. Most importantly, and in contrast to early reports, around 30% of patients have no identifiable trigger [8], demonstrating that a trigger is not required to diagnose TS. This is also reflected by the current InterTAK Diagnostic Criteria. Comprehensive overviews of reported triggers of TS have been published elsewhere [53, 54]. The frequently reported triggers may at least in part be subject to a reporting bias: Many patients with acute cardiovascular conditions such as hypertensive emergency, intracranial hemorrhage, or myocardial infarction also experience preceding triggers [55]. In addition, emotional or physical stress alone is probably not sufficient to induce TS: Given the abundance and currently high level of emotional stress in everyday life, the incidence of TS is inadequately low. One study concluded that the preexisting personality in TS patients provides susceptibility to TS, suggesting that coping mechanisms and an individually increased response to stress finally drive the onset of TS [56]. However, TS development in nonawake patients is not sufficiently explained by a psychosomatic model. The interplay between triggers, the adrenergic system, and the cardiac phenotype as a “response” are likely too complex for a simple linear explanation.

## Coexisting coronary artery disease

From the beginning, TS has been associated with the absence of coexisting coronary artery disease (CAD). In the first version of the Mayo Clinic Diagnostic Criteria, the presence of CAD with relevant stenoses was a rule-out criterion for the diagnosis of TS. Since apical TS was the only known form of TS at that time, the presence of CAD especially in the left anterior descending artery (LAD) often resulted in diagnosing an ACS instead. Magnetic resonance imaging (MRI) was not routinely available, hence there was no reliable measure to distinguish between ACS and TS. The recent consensus diagnostic criteria for TS allow for diagnosing TS in the presence of CAD (please see below). In contrast to the initial view that TS and obstructive CAD are contradictory, available data suggest that CAD is not rare in TS patients (Table 3; [57–62]). However, published studies on CAD and TS are rather small, especially the investigated cohorts of patients with TS and coexisting obstructive CAD. Moreover, these studies reported only few details on the nature of CAD, and therefore further studies are urgently needed. Coronary imaging studies, using either intravascular ultrasound or optical coherence tomography, again demonstrated that CAD might be present, but conflicting findings regarding coronary culprit lesions do not allow for definitive conclusions [58, 63–65]. Furthermore, ACS and TS can coexist [66–72], thus making the correct diagnosis may sometimes be challenging, at least without cardiac MRI (see below).

**Table 3:** Takotsubo syndrome and coronary artery disease

Study	Year	Origin	TS patients	Normal coronary arteries	Nonobstructive CAD	Obstructive CAD
Haghi et al.^S19^	2007	Germany	4	–	–	4 (100%)
Winchester et al.^S20^	2008	USA	31	12 (38.7%)	10 (32.3%)	9 (29.0%)
Kurisu et al.^S21^	2009	Japan	97	Not reported	Not reported	10 (10.3%)
Hoyt et al.^S22^	2010	USA	97	16 (16.5%)	81 (83.5%)	13 (13.4%)
Haghi et al.^S23^	2010	Germany	10	5 (50.0%)	2 (20.0%)	3 (33.3%)
Pawlowski et al.^S24^	2010	Poland	14	–	14 (100%)	–
Delgado et al.^S25^	2011	USA	11	–	11 (100%)	–
Parodi et al.^S26^	2013	Italy	450	315 (70.0%)	92 (20.4%)	43 (9.6%)
Bill et al.^S27^	2017	Germany	114	Not reported	Not reported	22 (19.3%)

References to the tables are provided as electronic supplementary material

*CAD* coronary artery disease, *TS* Takotsubo syndrome

A retrospective angiographic study of 109 TS patients found that tortuosity of coronary arteries and a “wrap-around” LAD are significantly more prevalent in TS patients than in age- and gender-matched control patients [73]. The study has limitations since patients with TS and coexisting CAD were not included and the control group had no CAD. Nevertheless, it might be worth studying whether anatomical variants and morphological characteristics of coronary arteries play a role in TS, either in the pathogenesis or as a disease modifier.

A very characteristic angiographic finding in TS is coronary slow-flow, especially in the LAD in patients with apical TS [74]. This might be attributed to microvascular dysfunction [75, 76], and its extent potentially correlates with adverse prognosis [77].

## How to diagnose Takotsubo syndrome

Since 2003, several diagnostic criteria have been proposed (Table 4). Of these, the most widely used criteria have been the revised version of the Mayo Clinic Diagnostic Criteria, which were published in 2008 [25]. With different criteria considerable confusion and debate emerged, especially whether patients with CAD, pheochromocytoma, without triggers, and without ECG changes should be classified as having TS [78–80]. The InterTAK diagnostic criteria ([7]; Table 5) are the most recent criteria, were agreed upon by most international leading TS investigators, and are largely congruent with the criteria published by the Taskforce on TS of the Heart Failure Association of the European Society of Cardiology [81]. The InterTAK Criteria further represent a modified and more precise version of the revised Mayo Clinic Diagnostic Criteria [25].

**Table 4:** Takotsubo syndrome diagnostic criteria

Year	Publication	Criteria
2003	Abe et al.^S28^	Abe criteria
2004	Bybee et al.^S29^	Mayo Clinic Diagnostic Criteria
2007	Kawai et al.^S30^	Japanese diagnosis guidelines
2008	Prasad et al.^S31^	Mayo Clinic Diagnostic Criteria, revised
2011	Omerovic^S32^	Gothenburg criteria
2012	Wittstein^S33^	Johns Hopkins criteria
2013	Redfors et al.^S34^	Gothenburg criteria, revised
2014	Parodi et al.^S35^	Takotsubo-Italian Network Proposal
2014	Madias^S36^	Madias criteria
2014	Redfors et al.^S37^	Gothenburg criteria, proposed new criteria
2016	Lyon et al.^S38^	ESC Heart Failure Association Taskforce Criteria
2018	Ghadri et al.^S39^	InterTAK Criteria

References to the tables are provided as electronic supplementary material

*ESC* European Society of Cardiology

**Table 5:** 2018 InterTAK Diagnostic Criteria for Takotsubo Syndrome

1.	Transient LV dysfunction^a^ (hypokinesia, akinesia, or dyskinesia) presenting as apical, midventricular, basal, or focal ballooning. RV involvement can be present. Transitions between types can exist. Wall motion abnormalities usually extend beyond the perfusion territory of a single epicardial coronary artery, but rare exceptions can exist (focal TS)
2.	Emotional and/or physical triggers may precede onset of TS (not mandatory)
3.	(Acute) neurologic disorders or pheochromocytoma may serve as triggers for TS
4.	New ECG abnormalities (ST-segment elevation, ST-segment depression, T‑wave inversion, and QTc prolongation). Rare cases without any ECG changes exist
5.	Moderate elevation of troponin and creatine kinase and commonly significant elevation of BNP
6.	Presence of CAD does not preclude diagnosing TS
7.	Patients have no evidence of infectious myocarditis^b^
8.	Postmenopausal women are predominantly affected

InterTAK Criteria shortened and adapted from the International Expert Consensus Document on Takotsubo Syndrome (Part I)^S39^

References to the tables are provided as electronic supplementary material

*CAD* coronary artery disease, *LV* left ventricular, *RV* right ventricular, *TS* takotsubo syndrome

^a^Documentation of recovery of wall motion abnormalities is required for diagnosing TS, however wall motion abnormalities may remain for a prolonged period of time. Death before documentation of recovery is an important exception, in this case TS may be diagnosed also without documentation of recovery

^b^Cardiac magnetic resonance imaging is recommended to exclude infectious myocarditis and confirm diagnosis of TS

**Fig. 2:**
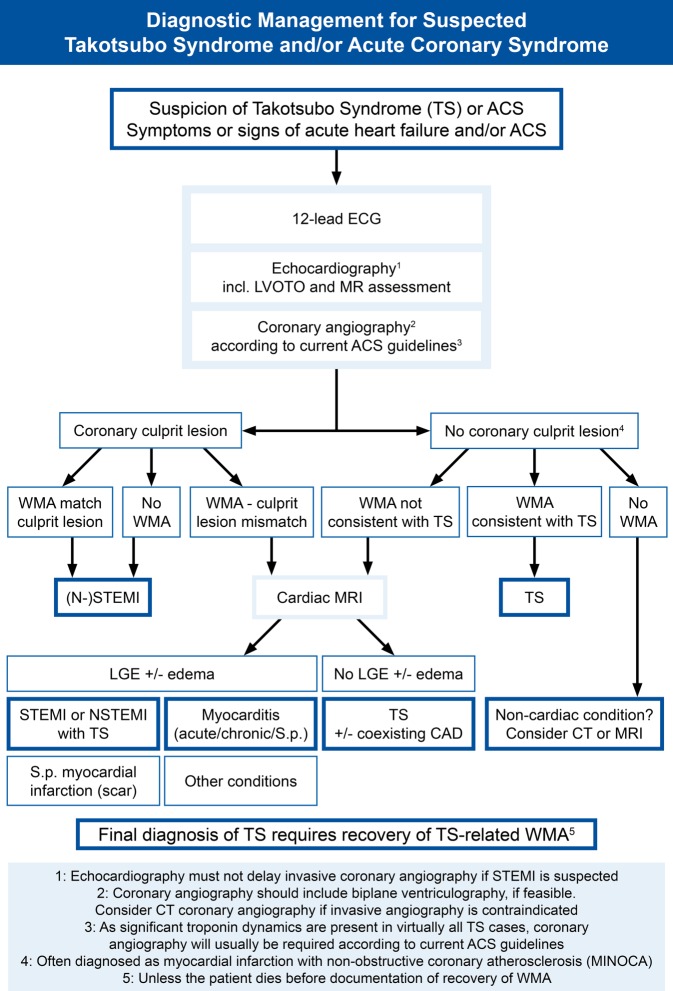
Diagnostic management algorithm for suspected takotsubo syndrome (*TS*) and/or acute coronary syndrome (*ACS*). *LGE* late gadolinium enhancement, *LVOTO* left ventricular outflow tract obstruction, *MINOCA* myocardial infarction with nonobstructive coronary atherosclerosis, *MR* mitral regurgitation, *MRI* magnetic resonance imaging, *S.p.* status post, *WMA* wall motion abnormalities

In clinical practice, it is often easy to diagnose TS, e.g., when a 70-year-old female patient with chest pain and ST-segment elevation undergoes coronary angiography, with angiographic absence of CAD and a typical apical ballooning pattern of the LV with basal hypercontraction. If wall motion abnormalities recover, TS can be finally diagnosed. However, frequently TS is suspected in patients with CAD or secondary to another severe comorbidity. Furthermore, it may at times be difficult to distinguish TS from myocarditis, ACS, or myocardial infarction with nonobstructive coronary atherosclerosis (MINOCA). Therefore, a pathway of meaningful clinical and technical investigation is required beyond diagnostic criteria, in order to make the correct diagnosis. Here, we propose a diagnostic pathway (Fig. 2), which covers the most important differential diagnoses to TS. Echocardiography is a cornerstone during diagnostic assessment, for which TS-specific aspects and recommendations have been published [82].

Echocardiographic examination should include assessment of LVEF, longitudinal strain, and wall motion abnormalities, in order to determine the TS type and the extent of LV dysfunction. Especially the focal type, which typically involves antero- or posterolateral segments of the LV, easily escapes echocardiographic standard views. Furthermore, echocardiography should screen for potential complications of TS, such as LV outflow tract obstruction (LVOTO), mitral regurgitation, and LV thrombus (Table 6).

**Table 6:** Echocardiography in Takotsubo syndrome

Parameters and potential pathological findings	
LV	Systolic function, semiquantitative assessment
	LVEF (%)
	Wall motion abnormalities, TS type
	LV thrombus
	E/e’ (estimate of diastolic function)
	Global longitudinal strain
	LVOTO (mean gradient, mmHg)
RV	Systolic function, semiquantitative assessment
	RV involvement
Mitral regurgitation	
SAM phenomenon of the anterior mitral leaflet	
Coronary flow in the distal LAD^a^	
Ventricular septal rupture	
Ventricular wall rupture	
Pericardial effusion	
Pleural effusion	

Adapted from a more detailed description of echo findings and parameters^S40^

References to the tables are provided as electronic supplementary material

*LAD* left anterior descending coronary artery, *LV* left ventricle, *LVEF* LV ejection fraction, *LVOTO* LV outflow tract obstruction, *RV* right ventricle, *SAM* Systolic anterior movement, *TS* Takotsubo syndrome

^a^Presence of flow in the distal LAD on echocardiography is not a sufficient substitute of coronary angiography, but is of interest in patients without ST-segment elevations and apical ballooning

Coronary angiography should be performed according to current ACS guidelines [83, 84]. Patients with TS usually present acutely with symptoms of ACS, and approximately 50% of patients have ST-segment elevation on ECG. In addition, patients admitted to hospitals for noncardiac reasons, who develop TS during hospitalization, are often identified by signs or symptoms of heart failure and shock or by arrhythmia or cardiac arrest. Nearly all patients finally diagnosed with TS have a significant rise in troponin levels, and coronary angiography would therefore be indicated according to current ACS guidelines. Thus, there are good reasons for invasive angiography in adult patients suspected of suffering from TS, even if coronary computed tomography is sometimes recommended in patients with suspected TS. Today, many interventional cardiologists do not routinely perform ventriculography in patients with suspected ACS. Of note, there are no robust data demonstrating that ventriculography is unsafe, unless performed in predictably dangerous scenarios such as LV thrombus, very high LVEDP, infective aortic valve endocarditis, or severe renal failure. In contrast, ventriculography offers a unique chance to investigate LV wall motion and its relation to coronary anatomy and pathology [85]. Furthermore, in clinical practice there might be a delay between admission and echocardiography, and TS wall motion abnormalities may have already partly recovered after 2–3 days [86]. Thus, operators who continue to employ ventriculography (unless contraindicated) might see more cases of TS in patients with signs and symptoms of ACS, and are further able to measure end-diastolic LV pressure and a potential LV outflow tract gradient early.

In many patients with suspected TS, differential diagnoses such as ACS or myocarditis cannot be finally ruled out by echocardiography and coronary angiography findings. This is the case in patients where wall motion abnormalities are rather congruent with the perfusion territory of a diseased coronary artery, or in patients where wall motion is abnormal in posterolateral segments, where myocarditis frequently manifests. In these cases, cardiac MRI adds essential information for making the correct diagnosis [87], as all the aforementioned major cardiac conditions are associated with distinct findings in cardiac MRI [27, 88–90]. Although not routinely used in all cardiac intervention centers, the availability and use of cardiac MRI will strongly increase over the next few years, and thereby contribute to enhancing accuracy and to avoiding misdiagnosis of cardiac conditions.

Most importantly, recovery of TS-related wall motion abnormalities is essentially required to finally diagnose TS, and especially in patients without early recovery, cardiac MRI should be considered.

## Incidence

TS is probably still an underdiagnosed condition. An estimated 1–3% of patients presenting to hospitals with ACS symptoms are finally diagnosed with TS. However, with dedicated imaging early after admission the number of diagnosed TS cases is probably higher, since in some patients recovery of wall motion abnormalities may occur before echocardiography is performed. Therefore, it is very important to search for wall motion abnormalities especially in patients presenting as ACS but without coronary culprit lesions (Fig. 2), with the intention not to miss underlying TS. However, TS may also concur in patients with a major leading cardiovascular condition such as myocardial infarction or pulmonary embolism [66–68, 70, 91, 92]. Thus, careful imaging is required also in presumably “clear” cases. Furthermore, TS might be an explanation for some unexplained cases of sudden death [93], but it is likely difficult to make a postmortem diagnosis [94].

Since the onset of TS is frequently associated with preceding stress from various sources, it appears reasonable that critically ill patients develop TS as a secondary disease. Indeed, the incidence of TS in critical care units in observational studies is substantial and higher than expected [95–98]. Therefore, TS should be suspected in patients with unexplained heart failure, hypotension, shock, arrhythmia, or troponin elevation, and dedicated echocardiography should be performed. In the acute phase differential diagnosis should also take into account myocarditis and MINOCA (Fig. 2), both of which are likely underdiagnosed, too, and may require MRI or endomyocardial biopsy for making a definitive diagnosis.

## Types of TS

**Fig. 3:**
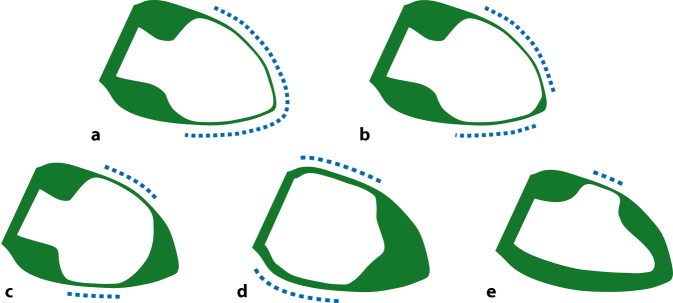
Takotsubo syndrome (TS) types. Schematic illustration of wall motion abnormalities, with *dashed lines* indicating stunned myocardium and *green areas* (hyper-)contractile myocardium. Right anterior oblique view as in standard ventriculography. **a** Apical TS, **b** apical TS with “nipple sign”, **c** midventricular TS, **d** basal TS, **e** focal TS

The “classic” and most frequent TS type is LV apical ballooning, which is characterized by midventricular and apical stunning, accompanied by basal hypercontraction (Fig. 3). Sometimes a tiny strictly apical portion of the LV is hypercontractile in apical TS, which has been referred to as the apical “nipple” sign [99]. Observing an apical nipple sign is helpful if TS needs to be distinguished from suspected transient LAD occlusion, since a positive nipple sign makes the latter very unlikely, if the LAD perfuses the hypercontractile nipple. There is no clear agreement on how to distinguish apical TS with an apical nipple sign from midventricular TS, if the nipple sign comprises a larger apical area.

Nonapical TS types have been described, which account for an estimated 25–30% of cases and may be more frequently diagnosed in the future since awareness of atypical TS types is increasing. The midventricular TS type is characterized by midventricular stunning with basal and apical hypercontraction [100–103], and is likely more frequent than currently assumed [104]. The basal type appears like a counterpart to the apical type, with basal stunning and midventricular and apical hypercontraction [105, 106]. The focal type occurs with focal stunning, most frequently in anterolateral or posterolateral segments [107, 108], and is often found with the most preserved LVEF and lowest complication rate of all types [104]. According to data from the InterTAK Registry, the TS type is not associated with clinical outcomes after adjusting for confounders [109]. However, there is a trend toward a worse prognosis with apical and basal and a better outcome with focal TS, respectively. There has been some confusion about the terms “inverse” and “reverse” TS, with the former describing basal and the latter describing midventricular TS. These terms should be avoided, as they are not unequivocal. Instead, basal and midventricular TS are the preferred designations. Basal TS is a common type in patients with subarachnoid hemorrhage, pheochromocytoma, and catecholamine administration. Therefore, such deleterious triggers should be ruled out once basal TS is diagnosed. Many cases of midventricular TS were published in the context of acute pulmonary triggers. Interestingly, in the literature nearly all children had an apical TS type (Table 2), the reason for which remains unknown. Recently, a fifth variant of TS was proposed, which is characterized by midventricular hypercontraction and basal and apical stunning [110]. However, confirmation of this phenotype by other cases and additional cardiac imaging is needed.

Right ventricular (RV) involvement is present in about 15% of cases [82], and isolated RV TS has been reported [111–113]. In clinical practice, the RV is unfortunately still somewhat neglected and RV dysfunction is underestimated. RV failure carries a profound risk for patients with heart failure, also in TS [114]. In the context of TS, RV failure is understudied and frequently overlooked. It is especially unknown whether all TS types have the same frequency of RV involvement, and whether RV involvement mirrors the types of LV involvement. Generally, once TS is suspected in a patient, the echocardiographic examination should also investigate the RV in particular (Table 6).

## In-hospital complications

Early reports suggested that TS is a transient disease with a very favorable prognosis. Indeed, some patients are admitted with severe chest pain and have rather severe LV dysfunction, but see a rapid recovery of LV function without arrhythmia, shock, or other complications. This is frequently the case in older female patients with emotional triggers, but only reflects a smaller part of the large spectrum of TS. In fact, the in-hospital phase is characterized by severe and frequent complications, which has been demonstrated by many groups from different continents [4, 9, 115, 116]. Beyond subgroups defined by age, trigger, TS type etc., a classification of primary and secondary TS has been proposed [81, 117], to account for differences in management and prognosis. Of note, patients admitted with ACS symptoms and finally diagnosed with TS (primary TS) have a better outcome than patients admitted for other reasons, in whom TS occurs later during hospitalization (often secondary TS; [118]). Thus, awareness should not only be directed at diagnosing TS in patients with ACS symptoms, but also in patients hospitalized for other reasons, who later develop signs or symptoms of TS such as chest pain, heart failure, shock, arrhythmia, ECG signs of ischemia, or syncope. In addition, in-hospital monitoring should not be omitted in both primary and secondary TS, as the incidence of acute complications is comparable to those with acute myocardial infarction [119]. If a patient with signs and symptoms of ACS undergoes coronary angiography and is diagnosed with apical TS instead, discharge on the next day (because “it is only TS”) is very likely too early and carries significant risk. The incidence of shock, resuscitation, and death is comparable to that in age- and gender-matched patients with ACS [4]. Selected patients might be candidates for a wearable defibrillator until recovery of LV wall motion abnormalities [120–122].

**Fig. 4:**
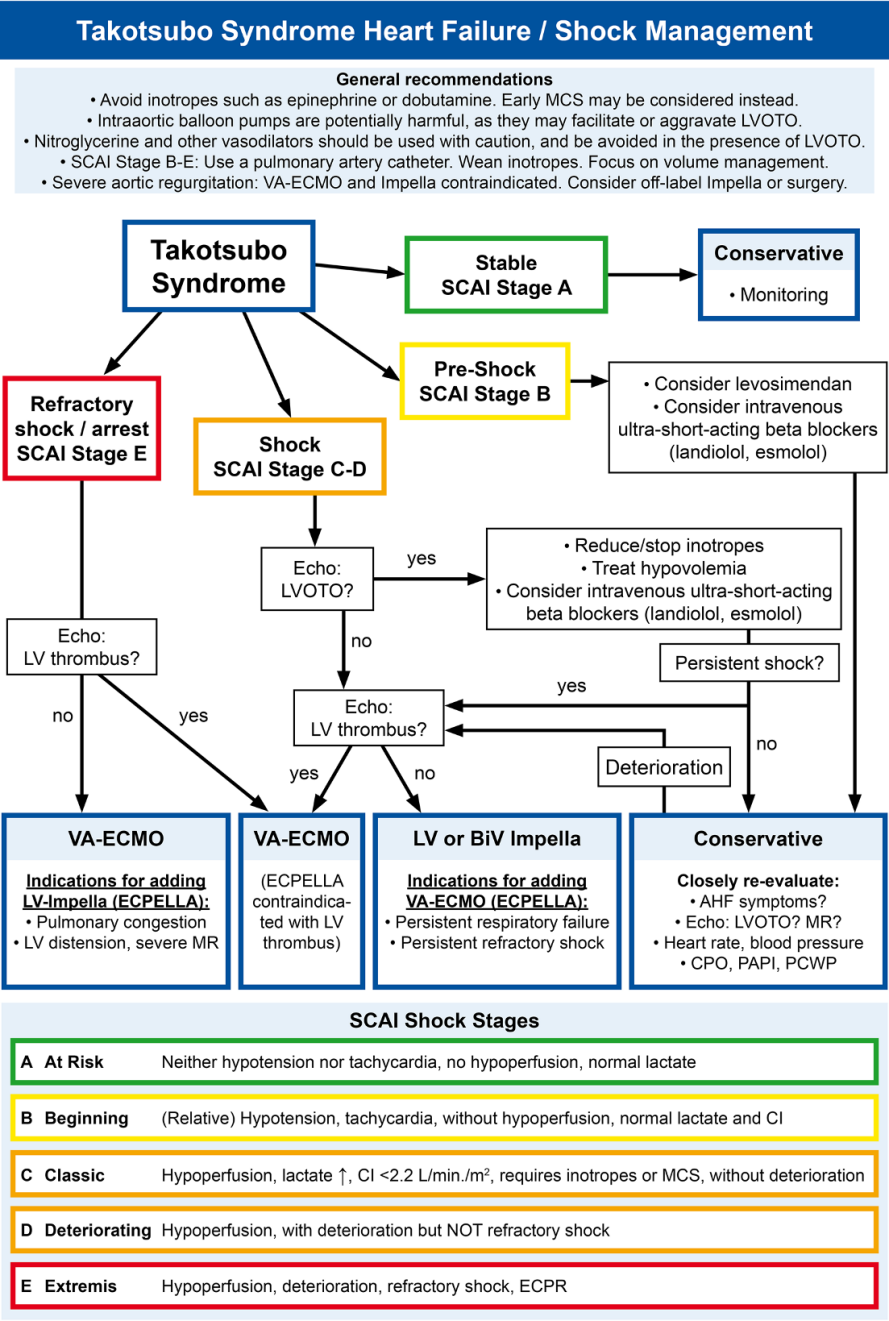
Takotsubo syndrome heart failure and shock management algorithm. SCAI shock stages adapted from Baran et al. [136] and Jentzer et al. [137]. *AHF* Acute heart failure, *BiV* biventricular, *CI* cardiac index, *CPO* cardiac power output, *ECPELLA* VA-ECMO combined with Impella, *ECPR* extracorporeal cardiopulmonary resuscitation, *LV* left ventricle, *LVEDP* left ventricular end-diastolic pressure, *LVOTO* left ventricular outflow tract obstruction, *MCS* mechanical circulatory support, *MR* mitral regurgitation, *PAPI* pulmonary artery pulsatility index, *PCWP* pulmonary capillary wedge pressure, *SCAI* Society for Cardiovascular Angiography and Interventions, *TS* takotsubo syndrome, *VA-ECMO* veno-arterial extracorporeal membrane oxygenation

Cardiogenic shock occurs in around 10% of patients with TS. However, in comparison to shock from myocardial infarction or myocarditis, some specific characteristics are present: Given the special role of catecholamines during pathogenesis, catecholamines and especially inotropes should be strictly avoided. Thus, mechanical circulatory support (MCS) may be considered earlier than in other conditions. There are no prospective studies on the use of MCS in TS patients with shock. Based on clinical experience with MCS devices in cardiogenic shock from other causes and integrating pathophysiological considerations, a proposal for heart failure and MCS management in TS patients is provided in Fig. 4. This proposal emphasizes to identify developing shock early, with the intention to prevent full development of the shock spiral.

## Recurrence and long-term risk

The first report on recurrence of TS was published in 2006 in Japanese [123]. In this report, the patient initially had midventricular TS, and subsequent recurrences were midventricular and biventricular apical TS. Later on, several cases with varying types in men and women across all ages were published (Table 7; [124]). One report hypothesized that recurring TS manifests in a different myocardial region than the first episode, i.e., that TS would protect against recurrence in the same region [125]. However, many reports of recurrence in the same region (Table 7) strongly oppose this hypothesis. On the other hand, numerous reports with different TS types in the same patient (Table 7) render the beta-receptor concept of TS development rather unlikely, at least as the only responsible step in pathogenesis. Overall, there is recurrence with all TS types across all ages in men and women, with a mean recurrence rate in adults of around 1.5–2% per year [124]. As extrapolated from case reports, patients with pulmonary triggers from chronic lung disease, with diarrhea or electrolyte disorders, and with drug or substance abuse tend to have a higher chance of recurrence.

**Table 7:** The spectrum of takotsubo syndrome recurrence

Study	Year	Sex, age at 1st episode	Number of recurrences	First episode	Gap	Second episode	Gap	Third episode	Gap	Fourth episode
Sharath Babu et al.^S41^	2019	Female, 59 years	1	Apical	8 months	Apical	–	–	–	–
Napp et al.^S42^	2015	Female, 63 years	1	Apical	2 years	Apical	–	–	–	–
Srivastava et al.^S10^	2016	Male, 14 years	1	Apical	6 months	Apical	–	–	–	–
Eitel et al.^S43^	2014	Female, 96 years	1	Apical	2 years	Midventricular	–	–	–	–
Xu et al.^S44^	2014	Female, 52 years	1	Apical	11 years	Midventricular	–	–	–	–
Rashed et al.^S45^	2019	Female, 43 years	1	Apical	8 months	Basal	–	–	–	–
Kato et al.^S46^	2014	Female, 65 years	1	Midventricular	3 years	Midventricular	–	–	–	–
Wever-Pinzon et al.^S47^	2011	Female, 82 years	1	Midventricular	4 months	Apical	–	–	–	–
Blessing et al.^S48^	2007	Male, 70 years	1	Basal	3 months	Apical	–	–	–	–
Binaghi et al.^S49^	2018	Female, 67 years	1	Basal	≈2 weeks	Apical	–	–	–	–
Piranavan et al.^S50^	2019	Female, 66 years	1	Focal	3 years	Midventricular	–	–	–	–
Chandorkar et al.^S51^	2014	Male, 28 years	1	RV (apical)	5 months	RV (apical)	–	–	–	–
Joe et al.^S52^	2013	Female, 83 years	1	RV (apical)	1 week	Apical	–	–	–	–
Luo et al.^S53^	2019	Male, 52 years	1	Biventricular (apical)	2 months	Biventricular (apical)	–	–	–	–
Cattaneo et al.^S54^	2015	Male, 66 years	2	Apical	1 year	Apical	2 years	Apical	–	–
Sager et al.^S55^	2011	Female, 66 years	2	Midventricular	9 months	Midventricular	3 years	Midventricular	–	–
Shimizu et al.^S56^	2006	Male, 60 years	2	Midventricular	2 years	Midventricular	3 months	Biventricular (apical)	–	–
Mugnai et al.^S57^	2015	Female, 64 years	2	Apical	8 months	Basal	4 years	Basal	–	–
Rodriguez et al.^S58^	2014	Female, 56 years^a^	2	Type not reported	Gap not reported	Apical	2 months	Basal	–	–
Ghadri et al.^S59^	2012	Female, 65 years	2	Midventricular	8 years	Apical	1 year	Focal	–	–
Opolski et al.^S60^	2016	Female, 62 years	3	Apical	10 years	Apical	3 years	Apical	2 years	Apical
Kaushik et al.^S61^	2011	Female, 56 years	5	Six episodes of TS over 4 years, with at least one apical and one basal type, suspected to be triggered by cannabis abuse						
Chandy and Dawson^S62^	2019	Female, 48 years	5	Six episodes of TS over 33 years, with at least two apical types and one focal type						

All publications in this table reported recovery of systolic ventricular function between episodes of recurrence

References to the tables are provided as electronic supplementary material

*TS* takotsubo syndrome

^a^At the time of the second episode

Beyond recurrence, TS also carries a long-term mortality risk [126–128]. This is probably mainly due to noncardiac causes, and it remains incompletely understood whether long-term risk is due to TS or whether TS occurs in patients already at higher risk due to other causes.

Recently, it was reported that myocardial and systemic inflammation is present in the acute phase of TS and that subtle changes may persist in the heart, in part questioning the concept of transient myocardial dysfunction and recovery [129–133]. It is indeed not surprising that a cardiac condition that is characterized by severe stunning and significant troponin release evokes an inflammatory response, and that long-term sequelae such as diastolic dysfunction and microscopic fibrosis persist in the myocardium. Although the recent findings are of major importance for a better understanding of the disease, it remains unknown whether they translate into clinical outcomes.

## Long-term therapy

TS was initially described as a “stress cardiomyopathy” with an emotional trigger in early years. As outlined above, there is indeed a strong and somehow specific association between the beta-adrenergic system and TS. Interestingly, in an ex vivo model with induced pluripotent stem cell-derived cardiomyocytes from TS patients, there was increased beta-adrenergic activity and response to catecholamines [134], further confirming this association. All this strongly suggests that beta-blockers should be beneficial in TS patients. However, in the InterTAK Registry, 30% of all patients and 60% of patients with recurrent TS were on beta-blockers before TS onset. Most of these were β1-specific. This demonstrates that beta-blockers are not generally sufficient to prevent TS or TS recurrence [4, 124]. In a post hoc analysis of discharge medication, mortality was comparable between TS patients with and without beta-blockers at discharge [4], although this analysis had significant limitations. However, it remains unknown whether a TS episode carries a reduced risk of complications, heart failure, and death in patients on beta-blockers than in patients without. Perhaps the mere blockade of the receptor is not as important as modification of downstream signaling. In summary, although there are many associations, there are no data demonstrating that beta-blocker prescription is of any specific benefit in TS patients. Therefore, beta-blockers should not be given routinely after recovery, unless there is another indication for their use or a study demonstrates a benefit and thereby justifies treatment.

## Conclusion

Takotsubo syndrome (TS) occurs in a variety of phenotypes across all ages and genders, and is associated with substantial risk during the acute phase. Mechanical circulatory support is an emerging strategy for patients with TS and shock, in order to avoid catecholamines and inotropes in particular. Prospective studies are needed in this context, as well as for medical treatment of TS with the intention to prevent recurrence.

## Caption Electronic Supplementary Material

Supplementary References to Tables 1–6
