# Supplementary material for: Takotsubo syndrome: between evidence, myths, and misunderstandings
Source: Herz. 2020 Mar 23;45(3):252–66. doi: 10.1007/s00059-020-04906-2 (PMC7198647; doi:10.1007/s00059-020-04906-2)
Supplement: Supplementary file 1 — Supplementary References to Tables 1–6 [file 59_2020_4906_MOESM1_ESM.docx]

**TAKOTSUBO SYNDROME: Between EVIDENCE, myths and misunderstandings**

L. Christian Napp MD^§^ and Johann Bauersachs MD FAHA FESC FHFA

Department of Cardiology and Angiology

Hannover Medical School, Hannover, Germany

**§: Correspondence:**

L. Christian Napp MD

Department of Cardiology and Angiology

Hannover Medical School

Carl-Neuberg-Str. 1, 30625 Hannover

phone +49-511-532-2246

fax +49-511-532-5412

napp.christian@mh-hannover.de

# Supplementary References

[1] [2] [3] [4] [5] [6] [7] [8] [9] [10] [11] [12] [13] [14] [15] [16] [17] [18] [19] [20] [21] [22] [23] [24] [25] [26] [27] [28] [29] [30] [31] [32] [33] [34] [35] [36] [37] [38] [39] [40] [41] [42] [10] [43] [44] [45] [46] [47] [48] [49] [50] [51] [52] [53] [54] [55] [56] [57] [58] [59] [60] [61] [62]

S1. Sharkey SW, Lesser JR, Maron MS, Maron BJ (2011) Why not just call it tako-tsubo cardiomyopathy: a discussion of nomenclature. J Am Coll Cardiol 57 (13):1496-1497. doi:10.1016/j.jacc.2010.11.029

S2. Rozema T, Klein LR (2016) Takotsubo cardiomyopathy: a case report and literature review. Cardiol Young 26 (2):406-409. doi:10.1017/S1047951115001249

S3. Greco CA, De Rito V, Petracca M, Garzya M, Donateo M, Magliari F (2011) Takotsubo syndrome in a newborn. Journal of the American Society of Echocardiography : official publication of the American Society of Echocardiography 24 (4):471 e475-477. doi:10.1016/j.echo.2010.08.002

S4. Hernandez LE, Martinez Y, Chan KC (2010) Takotsubo cardiomyopathy: an unusual cardiomyopathy at an unusual age. Cardiol Young 20 (5):577-579. doi:10.1017/S1047951110000831

S5. Schoof S, Bertram H, Hohmann D, Jack T, Wessel A, Yelbuz TM (2010) Takotsubo cardiomyopathy in a 2-year-old girl: 3-dimensional visualization of reversible left ventricular dysfunction. J Am Coll Cardiol 55 (3):e5. doi:10.1016/j.jacc.2009.08.050

S6. Maruyama S, Nomura Y, Fukushige T, Eguchi T, Nishi J, Yoshinaga M, Kawano Y (2006) Suspected takotsubo cardiomyopathy caused by withdrawal of bupirenorphine in a child. Circ J 70 (4):509-511. doi:10.1253/circj.70.509

S7. Fabi M, Testa G, Gesuete V, Balducci A, Ragni L (2013) An unusual cardiomyopathy after physical stress in a child. Congenit Heart Dis 8 (2):E45-48. doi:10.1111/j.1747-0803.2011.00610.x

S8. Otillio JK, Harris JK, Tuuri R (2014) A 6-year-old girl with undiagnosed hemophagocytic lymphohistiocytosis and takotsubo cardiomyopathy: a case report and review of the literature. Pediatr Emerg Care 30 (8):561-565. doi:10.1097/PEC.0000000000000189

S9. Berton E, Vitali-Serdoz L, Vallon P, Maschio M, Gortani G, Benettoni A (2012) Young girl with apical ballooning heart syndrome. International journal of cardiology 161 (1):e4-6. doi:10.1016/j.ijcard.2012.03.012

S10. Srivastava NT, Parent JJ, Hurwitz RA (2016) Recurrent takotsubo cardiomyopathy in a child. Cardiol Young 26 (2):410-412. doi:10.1017/S1047951115001377

S11. Zalewska-Adamiec M, Bachorzewska-Gajewska H, Kralisz P, Talalaj M, Pryzmont M, Dobrzycki S (2018) Sudden cardiac arrest in the course of takotsubo syndrome in a 15-year-old girl. Postepy Kardiol Interwencyjnej 14 (3):318-319. doi:10.5114/aic.2018.78341

S12. Ohwada R, Hotta M, Kimura H, Takagi S, Matsuda N, Nomura K, Takano K (2005) Ampulla cardiomyopathy after hypoglycemia in three young female patients with anorexia nervosa. Intern Med 44 (3):228-233. doi:10.2169/internalmedicine.44.228

S13. Okwechime R, Hawatmeh S, Galustian S, Khurana D, Mir P, Mir T (2018) 120: A case of reverse Takotsubo cardiomyopathy in a 26-year-old woman resulting from K2 overdose. Critical Care Medicine 46 (1):43. doi:10.1097/01.ccm.0000528140.11464.86

S14. Singh A, Southwick F, Gumm E, Makar JS (2012) Exploring takotsubo cardiomyopathy in an elderly patient with acute anxiety attack. W V Med J 108 (5):14-17

S15. Xu RH, Yu DQ, Ma GZ, Cai ZX, Ni CM, Chen P, Zhu ZD, Luo YH, Zhu GH, Huang JQ (2012) Takotsubo cardiomyopathy in a 90-year-old Chinese man. Chin Med J (Engl) 125 (5):957-960

S16. Budnik M, Piatkowski R, Kochanowski J, Glowczynska R, Gorko D, Kowalik R, Pietrasik A, Opolski G (2015) The oldest patient with takotsubo cardiomyopathy. J Geriatr Cardiol 12 (5):588-589. doi:10.11909/j.issn.1671-5411.2015.05.014

S17. Zalewska-Adamiec M, Bachorzewska-Gajewska H, Tomaszuk-Kazberuk A, Nowak K, Drozdowski P, Bychowski J, Krynicki R, Musial WJ, Dobrzycki S (2016) Takotsubo cardiomyopathy: serious early complications and two-year mortality - a 101 case study. Neth Heart J 24 (9):511-519. doi:10.1007/s12471-016-0857-z

S18. Bonfanti L, Buratti S, Vignali L, Lippi G, Masini F, Bianconcini M, Cervellin G (2018) Takotsubo cardiomyopathy in an ultra-centenarian woman. Acta Biomed 88 (4):529-532. doi:10.23750/abm.v88i4.6653

S19. Haghi D, Papavassiliu T, Hamm K, Kaden JJ, Borggrefe M, Suselbeck T (2007) Coronary artery disease in takotsubo cardiomyopathy. Circ J 71 (7):1092-1094. doi:10.1253/circj.71.1092

S20. Winchester DE, Ragosta M, Taylor AM (2008) Concurrence of angiographic coronary artery disease in patients with apical ballooning syndrome (tako-tsubo cardiomyopathy). Catheterization and cardiovascular interventions : official journal of the Society for Cardiac Angiography & Interventions 72 (5):612-616. doi:10.1002/ccd.21738

S21. Kurisu S, Inoue I, Kawagoe T, Ishihara M, Shimatani Y, Nakama Y, Maruhashi T, Kagawa E, Dai K, Matsushita J, Ikenaga H (2009) Prevalence of incidental coronary artery disease in tako-tsubo cardiomyopathy. Coronary artery disease 20 (3):214-218. doi:10.1097/MCA.0b013e3283299260

S22. Hoyt J, Lerman A, Lennon RJ, Rihal CS, Prasad A (2010) Left anterior descending artery length and coronary atherosclerosis in apical ballooning syndrome (Takotsubo/stress induced cardiomyopathy). International journal of cardiology 145 (1):112-115. doi:10.1016/j.ijcard.2009.06.018

S23. Haghi D, Roehm S, Hamm K, Harder N, Suselbeck T, Borggrefe M, Papavassiliu T (2010) Takotsubo cardiomyopathy is not due to plaque rupture: an intravascular ultrasound study. Clin Cardiol 33 (5):307-310. doi:10.1002/clc.20747

S24. Pawlowski T, Mintz GS, Kulawik T, Gil RJ (2010) Virtual histology intravascular ultrasound evaluation of the left anterior descending coronary artery in patients with transient left ventricular ballooning syndrome. Kardiol Pol 68 (10):1093-1098

S25. Delgado GA, Truesdell AG, Kirchner RM, Zuzek RW, Pomerantsev EV, Gordon PC, Regnante RA (2011) An angiographic and intravascular ultrasound study of the left anterior descending coronary artery in takotsubo cardiomyopathy. Am J Cardiol 108 (6):888-891. doi:10.1016/j.amjcard.2011.05.012

S26. Parodi G, Citro R, Bellandi B, Del Pace S, Rigo F, Marrani M, Provenza G, Leoncini M, Salerno Uriarte JA, Bovenzi F, Bossone E, Tako-tsubo Italian N (2013) Tako-tsubo cardiomyopathy and coronary artery disease: a possible association. Coronary artery disease 24 (6):527-533. doi:10.1097/MCA.0b013e3283645c4e

S27. Bill V, El-Battrawy I, Schramm K, Ansari U, Hoffmann U, Haghi D, Kuschyk J, Borggrefe M, Akin I (2017) Coincidental coronary artery disease impairs outcome in patients with takotsubo cardiomyopathy. QJM 110 (8):483-488. doi:10.1093/qjmed/hcx035

S28. Abe Y, Kondo M, Matsuoka R, Araki M, Dohyama K, Tanio H (2003) Assessment of clinical features in transient left ventricular apical ballooning. J Am Coll Cardiol 41 (5):737-742. doi:10.1016/s0735-1097(02)02925-x

S29. Bybee KA, Kara T, Prasad A, Lerman A, Barsness GW, Wright RS, Rihal CS (2004) Systematic review: transient left ventricular apical ballooning: a syndrome that mimics ST-segment elevation myocardial infarction. Ann Intern Med 141 (11):858-865. doi:10.7326/0003-4819-141-11-200412070-00010

S30. Kawai S, Kitabatake A, Tomoike H, Takotsubo Cardiomyopathy G (2007) Guidelines for diagnosis of takotsubo (ampulla) cardiomyopathy. Circ J 71 (6):990-992. doi:10.1253/circj.71.990

S31. Prasad A, Lerman A, Rihal CS (2008) Apical ballooning syndrome (Tako-Tsubo or stress cardiomyopathy): a mimic of acute myocardial infarction. Am Heart J 155 (3):408-417. doi:10.1016/j.ahj.2007.11.008

S32. Omerovic E (2011) How to think about stress-induced cardiomyopathy?--Think "out of the box"! Scand Cardiovasc J 45 (2):67-71. doi:10.3109/14017431.2011.565794

S33. Wittstein IS (2012) Stress cardiomyopathy: a syndrome of catecholamine-mediated myocardial stunning? Cell Mol Neurobiol 32 (5):847-857. doi:10.1007/s10571-012-9804-8

S34. Redfors B, Shao Y, Omerovic E (2013) Stress-induced cardiomyopathy (Takotsubo)--broken heart and mind? Vasc Health Risk Manag 9:149-154. doi:10.2147/VHRM.S40163

S35. Parodi G, Citro R, Bellandi B, Provenza G, Marrani M, Bossone E, Tako-tsubo Italian N (2014) Revised clinical diagnostic criteria for Tako-tsubo syndrome: the Tako-tsubo Italian Network proposal. International journal of cardiology 172 (1):282-283. doi:10.1016/j.ijcard.2013.12.239

S36. Madias JE (2014) Why the current diagnostic criteria of Takotsubo syndrome are outmoded: a proposal for new criteria. International journal of cardiology 174 (3):468-470. doi:10.1016/j.ijcard.2014.04.241

S37. Redfors B, Shao Y, Lyon AR, Omerovic E (2014) Diagnostic criteria for takotsubo syndrome: a call for consensus. International journal of cardiology 176 (1):274-276. doi:10.1016/j.ijcard.2014.06.094

S38. Lyon AR, Bossone E, Schneider B, Sechtem U, Citro R, Underwood SR, Sheppard MN, Figtree GA, Parodi G, Akashi YJ, Ruschitzka F, Filippatos G, Mebazaa A, Omerovic E (2016) Current state of knowledge on Takotsubo syndrome: a Position Statement from the Taskforce on Takotsubo Syndrome of the Heart Failure Association of the European Society of Cardiology. Eur J Heart Fail 18 (1):8-27. doi:10.1002/ejhf.424

S39. Ghadri JR, Wittstein IS, Prasad A, Sharkey S, Dote K, Akashi YJ, Cammann VL, Crea F, Galiuto L, Desmet W, Yoshida T, Manfredini R, Eitel I, Kosuge M, Nef HM, Deshmukh A, Lerman A, Bossone E, Citro R, Ueyama T, Corrado D, Kurisu S, Ruschitzka F, Winchester D, Lyon AR, Omerovic E, Bax JJ, Meimoun P, Tarantini G, Rihal C, S YH, Migliore F, Horowitz JD, Shimokawa H, Luscher TF, Templin C (2018) International Expert Consensus Document on Takotsubo Syndrome (Part I): Clinical Characteristics, Diagnostic Criteria, and Pathophysiology. Eur Heart J 39 (22):2032-2046. doi:10.1093/eurheartj/ehy076

S40. Citro R, Lyon AR, Meimoun P, Omerovic E, Redfors B, Buck T, Lerakis S, Parodi G, Silverio A, Eitel I, Schneider B, Prasad A, Bossone E (2015) Standard and advanced echocardiography in takotsubo (stress) cardiomyopathy: clinical and prognostic implications. Journal of the American Society of Echocardiography : official publication of the American Society of Echocardiography 28 (1):57-74. doi:10.1016/j.echo.2014.08.020

S41. Sharath Babu NM, Chacko ST, Chacko BR, Irodi A (2019) Recurrent Takotsubo cardiomyopathy in a postmenopausal Indian lady: Is there a pattern? J Postgrad Med 65 (2):112-115. doi:10.4103/jpgm.JPGM_383_17

S42. Napp LC, Ghadri JR, Bauersachs J, Templin C (2015) Acute coronary syndrome or Takotsubo cardiomyopathy: The suspect may not always be the culprit. International journal of cardiology 187:116-119. doi:10.1016/j.ijcard.2015.03.255

S43. Eitel I, Möller C, Graf T, Thiele H (2014) Recurrence of takotsubo cardiomyopathy with different ballooning patterns. International journal of cardiology 177 (1):25-26. doi:10.1016/j.ijcard.2014.09.043

S44. Xu B, Williams PD, Brown M, Macisaac A (2014) Takotsubo cardiomyopathy: does recurrence tend to occur in a previously unaffected ventricular wall region? Circulation 129 (7):e339-340. doi:10.1161/CIRCULATIONAHA.113.007015

S45. Rashed A, Shokr M, Subahi A, Siddiqui F, Alkatib A, Afonso L (2019) Reverse Takotsubo Cardiomyopathy in a Patient With Prior Apical Takotsubo Cardiomyopathy: Challenging the Beta Receptor Gradient Theory. Ochsner J 19 (3):256-259. doi:10.31486/toj.18.0027

S46. Kato K, Sakai Y, Ishibashi I, Kobayashi Y (2014) Recurrent mid-ventricular takotsubo cardiomyopathy. The international journal of cardiovascular imaging 30 (8):1417-1418. doi:10.1007/s10554-014-0469-x

S47. Wever-Pinzon O, Wever-Pinzon J, Tami L (2011) Recurrent Takotsubo cardiomyopathy presenting with different morphologic patterns. International journal of cardiology 148 (3):379-381. doi:10.1016/j.ijcard.2010.10.091

S48. Blessing E, Steen H, Rosenberg M, Katus H, Frey N (2007) Recurrence of takotsubo cardiomyopathy with variant forms of left ventricular dysfunction. Journal of the American Society of Echocardiography : official publication of the American Society of Echocardiography 20 (4):439 e411-432. doi:10.1016/j.echo.2006.10.021

S49. Binaghi G, Congia D, Cossa S, Ganga R, Giardina G, Matta G, Merella W, Melis M, Pasqualucci D, Serra E, Porcu M (2018) Seizures and recurrence of Takotsubo syndrome: One clinical presentation and trigger, but two different anatomical variants in the same patient. A case to meditate on. Seizure 63:37-39. doi:10.1016/j.seizure.2018.10.014

S50. Piranavan P, Kaur N, Shah N, Hannan J (2019) Forme Fruste in Recurring Mid-Ventricular Variant of Takotsubo Cardiomyopathy. Am J Case Rep 20:385-389. doi:10.12659/AJCR.915006

S51. Chandorkar A, Codolosa JN, Lippmann ML, Pressman GS, Sta Cruz JP (2014) Recurrent right ventricular takotsubo cardiomyopathy in a patient with recurrent aspiration. Echocardiography 31 (8):E240-242. doi:10.1111/echo.12686

S52. Joe BH, Hwang HJ, Park CB, Jin ES, Sohn IS, Cho JM, Kim CJ (2013) Takotsubo cardiomyopathy recurrence with left ventricular apical ballooning following isolated right ventricular involvement: A case report. Exp Ther Med 6 (1):260-262. doi:10.3892/etm.2013.1112

S53. Luo ELC, Kardos A (2019) Case report: recurrent biventricular Takotsubo cardiomyopathy in a middle-aged man with fatal outcome after full recovery. European Heart Journal - Case Reports 3 (4):1-6. doi:10.1093/ehjcr/ytz196

S54. Cattaneo M, Moccetti M, Pasotti E, Faletra F, Porretta AP, Kobza R, Gallino A (2015) Three Recurrent Episodes of Apical-Ballooning Takotsubo Cardiomyopathy in a Man. Circulation 132 (24):e377-379. doi:10.1161/CIRCULATIONAHA.115.017630

S55. Sager HB, Schunkert H, Kurowski V (2011) Recurrent mid-ventricular Tako-Tsubo cardiomyopathy: three episodes of a uniform cardiac response to varying stressors. International journal of cardiology 152 (1):e22-24. doi:10.1016/j.ijcard.2010.09.081

S56. Shimizu M, Kato Y, Masai H, Shima T, Miwa Y (2006) [Recurrent episodes of takotsubo-like transient left ventricular ballooning occurring in different regions: a case report]. J Cardiol 48 (2):101-107

S57. Mugnai G, Pasqualin G, Prati D, Menegatti G, Vassanelli C (2015) Recurrent multiform Takotsubo cardiomyopathy in a patient with epilepsy: Broken heart or brain? International journal of cardiology 201:332-335. doi:10.1016/j.ijcard.2014.11.212

S58. Rodriguez F, Nathan AS, Navathe AS, Ghosh N, Shah PB (2014) Serial classic and inverted pattern Takotsubo cardiomyopathy in a middle-aged woman. Can J Cardiol 30 (11):1462 e1467-1469. doi:10.1016/j.cjca.2014.04.002

S59. Ghadri JR, Jaguszewski M, Corti R, Luscher TF, Templin C (2012) Different wall motion patterns of three consecutive episodes of takotsubo cardiomyopathy in the same patient. International journal of cardiology 160 (2):e25-27. doi:10.1016/j.ijcard.2012.01.021

S60. Opolski G, Budnik M, Kochanowski J, Kowalik R, Piatkowski R, Kochman J (2016) Four episodes of takotsubo cardiomyopathy in one patient. International journal of cardiology 203:53-54. doi:10.1016/j.ijcard.2015.10.048

S61. Kaushik M, Alla VM, Madan R, Arouni AJ, Mohiuddin SM (2011) Recurrent stress cardiomyopathy with variable regional involvement: insights into etiopathogenetic mechanisms. Circulation 124 (22):e556-557. doi:10.1161/CIRCULATIONAHA.111.059329

S62. Chandy S, Dawson DK (2019) Lifelong recurrent takotsubo cardiomyopathy: a case report. European Heart Journal - Case Reports 3 (4):1-5. doi:10.1093/ehjcr/ytz191
